# Journeys to tuberculosis treatment: a qualitative study of patients, families and communities in Jogjakarta, Indonesia

**DOI:** 10.1186/1471-2458-9-158

**Published:** 2009-05-27

**Authors:** N Rintiswati, Y Mahendradhata, Suharna, Susilawati, Purwanta, Y Subronto, CM Varkevisser, MJ van der Werf

**Affiliations:** 1 Microbiology Department, Faculty of Medicine, Gadjah Mada University, Jogjakarta, Indonesia; 2 Public Health Department, Faculty of Medicine, Gadjah Mada University, Jogjakarta, Indonesia; 3 Epidemiology and Disease Control Unit, Public Health Department, Institute of Tropical Medicine, Nationalestraat 155, Antwerp, Belgium; 4 Jogjakarta Provincial Health Office, Jogjakarta, Indonesia; 5 Jogjakarta Municipality Health Office, Jogjakarta Indonesia; 6 Nursing Education Programme, Faculty of Medicine, Gadjah Mada University, Jogjakarta, Indonesia; 7 Internal Medicine Department, Faculty of Medicine, Gadjah Mada University, Jogjakarta, Indonesia; 8 Royal Tropical Institute, Amsterdam, The Netherlands; 9 KNCV Tuberculosis Foundation, The Hague, The Netherlands; 10 Center for Infection and Immunity Amsterdam (CINIMA), University of Amsterdam, Amsterdam, The Netherlands

## Abstract

**Background:**

Many tuberculosis (TB) patients in Indonesia are diagnosed late. We seek to document patient journeys toward TB diagnosis and treatment and factors that influence health care seeking behavior.

**Methods:**

TB patients in Jogjakarta municipality (urban) and Kulon Progo district (rural) were recruited from health care facilities participating in the DOTS strategy and health care facilities not participating in the DOTS strategy, using purposive sampling methods. Data were collected through in-depth interviews with TB patients and members of their family and through Focus Group Discussions (FGD) with community members.

**Results:**

In total, 67 TB patients and 22 family members were interviewed and 6 FGDs were performed. According to their care seeking behavior patients were categorized into National TB program's (NTP) dream cases (18%), 'slow-but-sure patients' (34%), 'shopaholics' (45%), and the NTP's nightmare case (3%). Care seeking behavior patterns did not seem to be influenced by gender, place of residence and educational level. Factors that influenced care seeking behavior include income and advice from household members or friends. Family members based their recommendation on previous experience and affordability. FGD results suggest that the majority of people in the urban area preferred the hospital or chest clinic for diagnosis and treatment of TB whereas in the rural area private practitioners were preferred. Knowledge about TB treatment being free of charge was better in the urban area. Many community members from the rural area doubted whether TB treatment would be available free of charge.

**Conclusion:**

Most TB patients took over a month to reach a DOTS facility after symptoms appeared and had consulted a number of providers. Their income and advice from household members and friends were factors that influenced their care seeking behavior most.

## Background

Indonesia's National TB Control Programme (NTP) faces many challenges despite its impressive performance in reaching the international targets for case detection (>70%) and treatment success (>85%) in 2006 [1]. The country still ranks third in the world in terms of tuberculosis (TB) burden and needs to ensure that it progresses beyond current achievements toward the Stop TB Partnership targets of halving prevalence and death by 2015 [1]. One key challenge for attaining these targets is early diagnosis and treatment of TB patients, which is considered to be the foundation of TB control. Delay in diagnosis and treatment is associated with greater transmission of infection [2] and poorer outcomes for the patients [3].

In Indonesia, the mean duration from start of the first TB symptoms to treatment is 63 days [4], which is on par with the 62 days reported for India [5] and surpasses the 30 days cut-off point commonly used to define long delay [6]. The NTP needs to be informed on the mechanisms underlying delays in receiving an appropriate diagnosis and treatment to devise remedial interventions. Studies from other countries have documented a wide range of factors associated with delay, including HIV infection, gender, rural residence, visits to private practitioners, low education, low awareness, self-treatment and stigma [6]. Little is known however about the importance of these factors in the Indonesian context. With this in mind, we seek to explore patient journeys toward TB treatment in Indonesia.

## Methods

### Study setting

Jogjakarta province is located in the central southern part of Java, Indonesia. The provincial administration is divided into five districts with a total population of about 3.2 million inhabitants. The province's primary care network consists of approximately 650 private practitioners and 118 community health centres. These first line services are backed up by 9 public hospitals and 24 private hospitals (including maternity and mental hospitals) as well as 5 chest clinics. The number of traditional healers practicing in the area is unknown.

The community health centres form the backbone of the DOTS (Directly Observed Treatment, Short-course) services in the province. The hospitals and chest clinics have also been engaged in the DOTS strategy through the Hospital DOTS Linkage project since the year 2000. Private practitioners (PPs) and traditional healers were not yet formally incorporated into the DOTS strategy at the time of the study. PPs diagnose TB based on clinical symptoms, chest X-ray or smear microscopy, traditional healers diagnose TB on symptoms. Some of the PPs refer their patients to DOTS services for diagnosis and treatment, albeit not systematically [7]. There are no institutional arrangements for DOTS yet for these care providers and patients have to pay for consultation and treatment.

### Study population and sampling

We used a purposive sampling method applying the extreme case sampling technique [8], selecting the two districts in Jogjakarta province with the lowest (Kulon Progo district) and highest (Jogjakarta municipality) case notification rate (CNR) in 2003, respectively 23/100,000 population and 110/100,000 population. The study population consisted of TB patients, their family and community members. Every individual diagnosed and recorded as TB patient by a public or private health care provider or a traditional healer was eligible for inclusion in the study. We aimed to recruit TB patients through formal and informal health care providers i.e. hospitals, community health centers, chest clinics, PPs, and traditional healers. During a three months data collection period we would be able to recruit 70 patients based on the number of patients detected in quarters before the data collection. These 70 patients were to be recruited using a purposive sampling method applying the maximum variation technique covering various types of care providers (Table 1) and weighting based on the ratio of smear-positive cases reported in Jogjakarta and Kulon Progo (11:3). The TB patient respondents were newly diagnosed or diagnosed a maximum of 3 months before the data collection. Additional inclusion criteria were local residency, clarity of registered home address and consent for interview. Patients meeting inclusion criteria within each care provider category were then recruited randomly. We halted the recruitment process when there were indications of information saturation.

**Table 1:** Sampling frame for recruitment of TB patients to be interviewed from different health care providers.

**Health care provider**	**Target**	**Number of patients reported during the data collection period**	**Number of patients consented for interview**	**Number of patients interviewed**
Hospital	10	25	25	18
Health centre	10	18	14	11
Chest clinic	10	36	36	29
Private practitioner	30	10	9	9
Traditional healer	10	-	-	-

**Total**	70	89	84	67

We aimed to interview 20 family members of interviewed patients who are most knowledgeable of the patients' experience, for DOTS patients this would be those who had been treatment observers. We asked all family members of interviewed patients whether they would be willing to be interviewed. From the list of consenting family members, we recruited family members using purposive sampling applying the maximum variation technique covering the various types of care providers and ratio of patients between Jogjakarta municipality and Kulon Progo district. We additionally considered the potential of the respective family members as respondents (communication ability and richness of information).

Community is defined as a group of people living in the smallest administrative area (hamlet). Community members living in the same hamlet as the included TB patients were eligible for inclusion in FGDs. The hamlets with a TB patient that qualified for participation in the focus group discussions (FGDs) were identified by the district health officer, as the FGDs were organized in conjunction with regular community meetings. Hence the FGD participants were attendants of the meetings who agreed to stay and participate in the FGD after the meeting.

### Data collection and analysis

NR, Sh, Su, Pu and YS performed in-depth interviews with the TB patients and with family members of TB patients. The guidelines for the in-depth interviews for patients included questions on age, sex, level of education, economic status [income more or less than standard minimum wage, i.e. IDR 400,000 (equivalent to US$ 47 per month in 2003)], occupation, history of the symptoms, history of their initial and subsequent health care seeking behavior and treatment, details of their management, knowledge and perception of TB, and perceived stigmatization. Interviews of family members included questions on perceptions of the patient's disease history and advice given to the patient.

NR, Sh, Su, Pu and YS also conducted six FGD with community members: three in the Jogjakarta Municipality (urban) and three in Kulon Progo District (rural). The guidelines for the FGD covered knowledge of the community on TB symptoms, cause(s), transmission, consequences, cure, treatment quality and cost, action taken in case a relative or neighbor would suffer from TB, and opinion of existing health care services.

The interviews and FGDs were conducted in the Indonesian language or the local Javanese dialect and recorded on audio-tape with permission from the subjects. The recording was transcribed within 48-hours by a research assistant and checked by at least one core research team member. The transcripts were scrutinized to identify emerging and recurrent themes and a codebook was progressively established and structured. Text units were coded systematically. Coding frequency permitted to identify key issues and trends. All of these analysis procedures were carried out in Indonesian language by at least two different persons within the research team. English translation was only carried out in the process of report writing. The original interviewers had the opportunity to make corrections to the translated quotations.

The study was approved by the ethical review committee of the Faculty of Medicine, Gadjah Mada University, Indonesia.

## Results

### TB patient perspective

#### TB patient characteristics

We interviewed 67 patients, 50 from Jogjakarta municipality, and 17 from Kulon Progo (Table 2). There were more male than female patients (3:2). The mean age of males was 42.5 years and of females 36.4. Most respondents (40.3%) had completed senior secondary education. We did not succeed in recruiting patients from traditional healers because it appeared that traditional healers did not keep a patient register from which TB patients can be selected.

**Table 2:** Characteristics of interviewed TB patients in Jogjakarta municipality and Kulon Progo District.

**Characteristic**	**Number of respondents (%)**		
	
	**Jogjakarta Municipality**	**Kulon Progo District**	**Total**
**Sex**			
Male	30 (60.0)	10 (58.8)	40 (59.7)
Female	20 (40.0)	7 (41.2)	27 (40.3)
**Age**			
15–24	13 (26.0)	1 (5.0)	14 (20.9)
25–34	12 (24.0)	1 (5.0)	13 (19.4)
35–44	13 (26.0)	7 (41.2)	20 (29.9)
45–54	6 (12.0)	3 (17.6)	9 (13.4)
55–64	4 (8.0)	3 (17.6)	7 (10.4)
≥ 65	2 (4.0)	2 (11.8)	4 (6.0)
**Educational background**			
No formal education	1 (2.0)	1 (5.9)	2 (3.0)
Elementary school	8 (16.0)	5 (29.4)	13 (19.4)
Junior high school	9 (18.0)	4 (23.5)	13 (19.4)
Senior high school	22 (44.0)	5 (29.4)	27 (40.3)
Vocational college	6 (12.0)	1 (5.9)	7 (10.4)
University	4 (8.0)	1 (5.9)	5 (7.5)
Income*			
< Standard minimum wage	10 (20.0)	7 (41.2)	17 (25.4)
≥ Standard minimum wage	40 (80.0)	10 (58.8)	50 (74.6)
**Recruiting facilities**			
Hospital	17 (34.0)	1 (5.8)	18 (26.9)
Health centres	6 (12.0)	5 (29.4)	11 (16.4)
Chest clinics	21 (42.0)	8 (47.1)	29 (43.3)
Private practitioners	6 (12.0)	3 (17.6)	9 (13.4)

**Total**	50 (100.0)	17 (100.0)	67 (100.0)

* Standard minimum wage refers to the minimum amount which must be paid monthly to a full-time employee for services he/she provide to the employer as determined by government regulation. The standard minimum wage for Jogjakarta province in 2004 was US$ 40.

#### Care seeking behavior patterns: the road to DOTS

The most common initial symptom reported by the TB patients was cough (51/67), frequently accompanied by other symptoms such as fever, chest discomfort, and cough with blood. Only two patients actually thought of TB early in their course of illness.

*"It was TB, no?" *(56 year-old, male, rural resident)

*"It was the 100-days cough *[Whooping cough] ..." (42 year-old, female, urban resident)

*"I was terrified. It could have been anything ... some kind of infection, or perhaps cancer *..." (44 year-old, male, urban resident)

The interviewed TB patients can be categorized into four care seeking categories (Table 3), ranging from those who reached the DOTS facility within one month after the start of symptoms (and consulted a maximum of one non-DOTS provider/applied self medication) to those who had not reached a DOTS facility at the time of interview. 'Slow-but-sure' patients and 'shopaholics' formed the biggest categories (23/67 and 30/67). Patients showed no apparent difference in care seeking behavior according to urban-rural residence, gender, and educational level. Male patients with a higher educational level were "shopping" as often as female patients with an elementary education background. The majority (15/17) of patients with a monthly income less than the standard minimum wage showed the 'NTP's dream' and the 'slow-but-sure case' type of care seeking behavior, while most (28/50) patients with an income higher than the standard minimum wage exhibited the 'shopaholic' and the 'NTP's nightmare case' behavior.

**Table 3:** Care seeking behavior types of TB patients in Jogjakarta municipality and Kulon Progo district.

**Behavior type**	**Description**	**n (%)**
The NTP's dream case	A TB patient who reached a DOTS facility within one month or less after start of first symptom, usually after applying self medication or consulting one non-DOTS provider	12 (17.9%)

The slow-but-sure case	A TB patient who took more than one month to reach DOTS facility after self-medication or consulting one non-DOTS provider	23 (34.3%)

The shopaholic case	A TB patient who took over one month to reach a DOTS facility and consulted two or more non-DOTS providers	30 (44.8%)

The NTP's nightmare case	A TB patient had not reached a DOTS facility at the time of interview	2 (3.0%)

TOTAL		67 (100.0%)

After the start of the TB symptoms most patients first applied self medication using over the counter drugs or traditional medicine before seeking help from a care provider. The first health care provider contacted by most (20/67) respondents was a private physician followed by health centre (17/67), chest clinic (10/67) or a private hospital (9/67). This pattern of care provider preference was most prominent in Jogjakarta municipality. In Kulon Progo district, the first choice was a health centre (5/17) while the private physician (4/17) and nurse/midwife (4/17) were second in rank. Most patients reached these first choice providers within less than one month after the start of the symptoms. The most frequently mentioned reason for choosing a private physician was proximity (8/16). Other reasons were previous favorable experiences, low cost, health insurance scheme appointments and perceived quality of the facility. Advice of others (parents, spouse or friends) played an important role in patients' choices. Most of the interviewed patients were positive about their initial provider.

*"People in my neighbourhood always go there *[private practice], *since we were young until we are old, we always go there" *(30 year-old, male, urban resident)

*"It's *[health centre]* the closest health care facility, very cheap, and easy transportation" *(52 year-old, male, rural resident)

Notably, most (38/67) patients were not examined by smear microscopy by the first provider they consulted. Accordingly, many (16/67) patients did not feel improvement after receiving treatment and eventually went for consultation to another provider. They finally reached a DOTS facility either through referral by the previous provider or by chance. Others stated that they came to a DOTS facility because they considered it a good facility according to their own experience or the experience of a relative or an acquaintance.

*"Then, the cough just didn't get any better, I kept coughing .... So I forced myself to go to the chest clinic" *(41 year old, male, urban resident)

*"Then I was advised by the specialist that there's a possibility of free treatment at the health centre, and he gave me a referral letter" *(45 year old, male, urban resident)

There were also self-transfers from DOTS services to non-DOTS services. These self-transfers were mainly due to absence of improvement of symptoms. Almost half of these group (7/16) reported that they had not been requested to provide a sputum sample at the DOTS services they first consulted.

*"I first went to a health centre, three times...then to a private practitioner twice, and then to another private practitioner once, but I didn't feel better...then somebody advised me to go for an x-ray at the chest clinic..." *(48 year old, female, urban resident)

#### Patients' knowledge of TB and TB case management

All respondents said they had received information on TB by their treatment provider. However, when asked about the cause of TB 16 respondents did not know the cause, 18 mentioned non-infectious causes such as smoking, alcohol, stress, fatigue, fried food, sleeping on the floor, staying up all night, and catching a cold, 7 patient did not provide an answer, and only 26 mentioned an infectious cause such as germs, virus or bacteria.

*"I don't know what caused this disease ..." *(45 year-old, female, urban resident)

*"... because I sleep on the floor .. no mattress, and sometimes no pillow ..." *(23 years old, male, urban resident)

*"Honestly, it's probably because I smoke ... I smoke a lot of cigarettes, which is why now I stop smoking ..." *(41 year-old, male, urban resident)

*"Maybe I got infected by someone, but I am not sure ... I went for a massage and he told me it's probably because I am too stressed for the coming exam" ...... *(18 year-old, female, urban resident)

Almost all respondents (57/67) said that TB was a transmissible disease, also those who said that TB was caused by non-infectious agents. Knowledge about the method of TB transmission was comparable among the four different types of health care seeking behavior groups. Most respondents mentioned that TB was transmitted by droplets, dust, cough, or through the air.

When the patients were asked how TB could be cured, most respondents with "NTP dream" or 'shopaholic' behavior said that TB could be cured if the patient would take the TB drugs regularly.

" [I can be cured if] *I stop smoking, don't stay up late at nights, take drugs regularly and comply *[to doctor's instructions]" ... (19 year old, male, urban resident)

#### Stigmatization of TB patients

Most patients (51/67), regardless of their educational background, reported that they did not feel stigmatized because either their spouse, relatives and friends were supportive or they showed no behavioral change.

*"Family and friends know, their behavior didn't change, all stays nice, no one avoids me..." *(19 year old, male, urban resident)

Only 7 patients mentioned that friends/neighbours seemed to avoid them.

*"Yes, some people avoid me, I don't know, probably they are afraid to get infected. Before we met often, now we don't ..." *(37 year-old male, rural resident)

Most patients (48/67) felt that TB patients do not need to be isolated. This perception is similar regardless of income and educational background. Still, in Kulon Progo, males were more comfortable informing their friends and neighbours regarding their illness than females. Stigmatization of TB patients by individuals in the patient's environment occurred particularly in Kulon Progo, although it did not seem to be very serious.

### Family member perspective

#### Family member characteristics

Of the 22 relatives of TB patients we interviewed, most (80.9%) were from Jogjakarta municipality, 19.1% came from Kulon Progo district. The distribution of age, income and education was comparable between respondents from the urban and the rural area.

#### Observations of family members

The most common symptom of TB that relatives noticed in TB patients was cough (18/21); other symptoms noticed were fever, chest discomfort and bloody cough. The respondents believed that their relative was suffering from lung disease, or dry cough or whooping cough.

*"I was wondering what my wife is suffering from...could it be typhoid? I had typhoid before...maybe it's typhoid, she is also difficult when it comes to eating" *(35 years-old, male, urban resident)

Only 3 respondents considered that their relatives' illness may be 'flek' (local term for TB).

*"Yes, I knew immediately, it was TB, no? Probably she got it from me, I am diabetic and have been treated for TB as well" *(55 years-old, male, urban resident)

#### Advice of family members

Next to all relatives (20/21) provided the patient with advice when the first symptoms appeared. Most (17/20) suggested the patient to first use over-the-counter-drugs or traditional medicine. The remaining few suggested going to a health provider directly. When symptoms persist or worsen then relatives advised patients to go to health provider. Private physicians were most often recommended by relatives (9/21) followed by private hospitals, chest clinics and health centers. Many (7/21) stated that they made their recommendation based on previous experience and affordability.

*"Yes, I advised him to consult the general practitioner whom we already know....because if he knows us, he is usually more attentive, no?" *(70 year-old, male, urban resident)

*"She came home from a funeral with a fever, so I took her to the private hospital where they gave her some drugs ... I am more confident with that private hospital; Every time any of my children falls ill I always take them there" *(46 year old, female, urban resident)

*"Well, that's clear. If we require treatment we should be efficient and go to the health centre. Going to the health centre is like saving energy if we talk about electricity...so that's an option...we also have to look at our budget" *(32 year-old, male, urban resident)

#### Stigma

Nearly all (20/21) the respondents felt fear, surprise, or were worried or upset when they learned that their relative was suffering from TB. Most (14/21) however were not scared of getting infected, nor did they feel the need for full isolation of the TB patient. More than half (12/21) saw the need to separate patients as far as eating, sleeping and cooking was concerned. Most spouses (9/14), in contrast to blood relatives, stated the need for separate sleeping, eating and cooking.

*"If he cooks for himself it's OK, perhaps .... For others no .... What if he sneezes, cough" *(40 year-old, wife of a TB patient, rural resident)

### Community perspective

#### Characteristics of FGD participants

The six focus group discussion (FGD) groups consisted of 5 to 8 participants (Table 4). Four groups contained only females and two groups only males. All groups had members with different educational and occupational background.

**Table 4:** Characteristics of participants of the focus group discussions in Jogjakarta municipality and Kulon Progo district.

**Group**	**Number**	**Sex**	**Mean age in years (range)**	**Education**	**Occupation**
Jogjakarta 1	6	Female	55.5 (46 – 62)	University graduate (3)	Gov. employee (3)
				Senior High School (3)	Housewife (3)

Jogjakarta 2	8	Female	52.0 (45 – 65)	Diploma (2)	Housewife (3)
				Senior High School (4)	Laborer (2)
				Junior High School (1)	Private sector (3)
				Elementary School (1)	

Jogjakarta 3	5	Male	37.6 (20 – 50)	Diploma (1)	Gov. employee (1)
				Senior High School (4)	Private sector (4)

Kulon Progo 1	5	Female	43.6 (35 – 60)	University graduate (2)	Gov. employee (2)
				Diploma (1)	Housewife (3)
				Elementary School (2)	

Kulon Progo 2	6	Male	44.5 (32 – 55)	Senior High School (2)	Farmer (3)
				Junior High School (1)	Laborer (2)
				Elementary School (3)	Seller (1)

Kulon Progo 3	6	Female	49.5 (30 – 63)	University Graduate (1)	Housewife (4)
				Senior High School (1)	Laborer (1)
				Junior High School (1)	Seller (1)
				Elementary School (3)	

#### Knowledge about TB

Only a few (8) FGD participants actually mentioned that TB is caused by bacteria. There was a wide variety of other responses in regard to the cause of TB, including mental stress, fatigue, dirty air, smoking, heredity and magic.

*" I have heard from a neighbor, from the radio, TV, that TB is inherited ....." *(34 year-old, male, urban resident)

*"Yes... in the old days people said that TB was caused by magic, but nowadays we don't often hear it... *[still] *yes, TB can be caused by magic" *(55 year-old, male, rural resident)

Nonetheless, all groups in Jogjakarta and in Kulon Progo knew that TB is infectious and most of the participants even seemed to know roughly how TB is transmitted.

*"....... Do not allow her/him to come to the community meeting *[while laughing].* It is difficult to avoid having contact with her/him*...." (35 year-old, university graduated, female civil servant, rural resident)

All the groups from Jogjakarta and two groups from Kulon Progo agreed that TB can be cured by regular treatment and follow up or by visiting a lung clinic. Members of one group from Kulon Progo however did not perceive TB to be curable by regular treatment. Their view on how to cure TB was that the patient should rest, relax, and be happy.

*"If he/she is free of stress and happy then he/she can be cured*..." (63 year-old, elementary school, female, rural resident)

#### Opinion about TB health services and TB treatment

All groups reported that they would recommend a TB patient to go to a qualified health care provider. FGD participants in Kulon Progo would recommend more frequently to visit a health center because the network of health centers was considered cheap and approachable. Private general practitioners or specialists would be recommended because these were considered the experts, available at any time especially in the evening when health centers were closed. Those who preferred the hospital had a preference for government hospitals above private ones because they considered government hospitals cheaper. Participants also valued hospitals because they believed the services to be fast and accurate. When asked what would be the best health care provider for themselves if they would have TB, participants from the urban area mostly chose the hospital (government and/or private) or chest clinic, but participants from the rural area preferred a private practitioner or internist.

*"...I choose a *[private] *doctor as the best care giver because in the health center the personnel would get mad at us if we come at the last minute even if the health center is still open*..." (60 year-old, female, housewife, rural resident)

Ideal services according to the participants in both areas were services that provide fast, cheap and accurate care, with appropriate medication. Most participants from the urban area knew which health care units provided free drugs for TB treatment. Some participants in rural areas were skeptical whether free treatment actually existed. They did not consider isolation of TB patients a necessity

## Discussion

Our study revealed that there are four basic types of TB patient journeys in the study area. The majority of the TB patients (55/67) were diagnosed quite late (delays of >30 days) and many took dubious treatment before TB diagnosis and initiation of appropriate (DOTS) treatment. Most patients and their family and community members were knowledgeable of the fact that TB is a transmissible disease and that it can be cured by taking drugs. There were notable misconceptions regarding the cause of TB. Stigma was present albeit limited. Gender, rural/urban residence and educational level made no apparent difference in care seeking behavior. Income/cost in connection with home-clinic distance, and advice from household members or friends seemed to be more influential.

The patient journey patterns suggest that only 12 patients in the study area were diagnosed by DOTS providers within one month after the first symptoms appeared. Delay for a month or more was found in TB patients in Lusaka [9] and South India [10] respectively. Surveys from Ethiopia and South Africa have reported longer patient delays [11,12]. Notably, in our study patients with a low income were diagnosed and treated earlier with DOTS than those with a higher income. This was not documented in the other studies. The observed differences reflect differences of contextual factors, most importantly access to general care and DOTS services of the poor, and stereotypes about what people consider high quality care which make them look for other forms of care if they have the means.

A considerable proportion of the interviewed TB patients had been in contact with non-DOTS providers before turning to DOTS services. Lonnroth et al. [13] reported similar findings in Vietnam where most TB patients preferred to seek care first at private physicians (17.9%) or private pharmacies (29.9%). A study in South India [10] showed that previous contact with private providers significantly contributed to greater health system delay. Notably, there is also a transfer from DOTS services to non-DOTS services, most likely due to failure to identify TB suspects, poor quality of the services or patients' over-expectation of a speedy recovery, as was observed in Vietnam and Bali [13,14].

Over 80% of the TB patients in the study areas consulted several providers, both DOTS and non-DOTS, before they were diagnosed. This is in line with the findings of a study in South India where only 22% of the patients were diagnosed at the health facility where they first sought care, the others shopped around for care at various health facilities before a diagnosis of tuberculosis was made [10]. Nearly half of all the patients in the study in South India had visited three or more health facilities before a diagnosis of tuberculosis was made. In our setting, shopping around appeared related to interrelated factors such as home-clinic distance, perceived quality, cost, previous experience and advice from others.

WHO recommends in the new Stop TB Strategy to engage non-NTP providers [15]. However, the evidence on the effectiveness of the PPM DOTS strategy remains controversial with wide variation of outcomes across settings [16,17]. Taking into account our study findings, i.e. the large number of TB patients seeking health care at private practitioners, assessment of the potential contribution of a PPM initiative in Indonesia is urgently needed to guide further program policy making.

We did not find evidence that educational level influenced care seeking behavior. Godfrey-Faussett et al. [9] reported a similar observation in their delay study in Zambia. Rajeswari et al. [10], however, showed that there is an association between patient delay and educational background in South India. There is a need to take into account the special role of TB knowledge in this association. Portero et al. [18], reported that knowledge of germs as the cause of TB showed a significant correlation with college level education, while belief of heredity as a cause was significantly associated with non formal education. Others reported that lack of knowledge about TB treatment and large home-clinic distances were factors associated with longer patient's delay in smear negative patients [10]. Another study by Portero et al. [18] showed that only 25% of those interviewed knew that TB is due to germs (bacteria or microbes). Watkins and Plant [14] reported that on the neighboring island of Bali people believe that TB is caused by magic, spirits or heredity so they didn't need to attend the health providers. We also found these beliefs regarding the cause of TB in our study population. It should be noted that almost all the interviewed patients had at diagnosis received health education on TB. Thus it is surprising that so many TB patients did not know the biomedical cause of TB. Contrary to the patients in Bali, however, they had found their way to biomedical care.

Like in Bali [14], community members related causes mostly to physical factors such as staying up late at night, sleeping on the floor, working too hard and psychological factors including mental stress and worry. These non-infectious causes were also mentioned in another study carried out in Manila [18]. Despite the varying causes mentioned, participants in our study agreed that TB is an infectious and transmissible disease. Ways of transmission they cited were by talking, coughing, by dirty air, by sputum, or by sharing eating utensils, as was found in the study in The Philippines [18].

Delay studies have reported conflicting patterns of gender differences. Rajeswari et al. [10] showed that, contrary to popular belief, in South India men rather than women were more likely to delay seeking care: 32% of the male TB patients waited longer than one month to seek care against 23% of the females. Our findings do not indicate that gender influences care seeking behavior. This however should be further substantiated by a quantitative study with a larger sample.

Income seems to be closely linked to care seeking actions, though these actions not necessarily lead to the most cost-effective care. Those with a higher income, for example, could access a wide range of (private) providers and regarded the quality of DOTS providers at the health centers as poor. In contrast, those with an income below the minimum wage tended to seek low cost public services, particularly at local health centers, though they also might have preferred private treatment. Our findings show that advice of relatives such as spouses, parents or friends played an important role in seeking initial care from particular providers. The most frequently mentioned reason for choosing an initial provider was advice from household or community members. Both family and community members confirmed that they often provided patients with advice. Thus, the opinion of household and community members is quite influential in regard to care seeking behavior.

Our findings indicated that most patients in our study area didn't feel stigmatized because either their spouse, relatives and friends were supportive, or they showed no change in behavior when it became known that a patient had TB. There were however indications that spouses stigmatize patients more than blood relatives. Godfrey-Faussett et al. [9] reported that in Zambia there was evidence of considerable stigmatization of TB patients by the community as well as by relatives. The limited magnitude of stigma we documented may reflect the general level of education and the intensity of health promotion campaigns related to TB.

We have collected data mainly on patients' related factors rather than providers'. However, our findings revealed that providers seem to contribute rather considerably to total delay by frequently failing to examine TB suspects with smear microscopy. The current body of evidence does point out that the solutions to decrease long delays to TB treatment are to be sought on the providers', not on the patients' side [19]. Thus, interventions to ensure more proper implementation of NTP guidelines in health facilities seemingly are urgently needed.

This study is limited by qualitative research boundaries. Issues perceived by patients, family members and communities were identified. Although trends emerge, the respective influence of each issue was not quantified. This could be documented through a quantitative survey building on our findings, which points out the key issues to be taken into account. We also could not include traditional healers due to the difficulty of identifying and approaching them as they were largely unregulated and not institutionalized. Additionally, with the few who were identified, we did not manage to establish sufficient rapport for recruiting their patients for interviews within the study data collection period or they did not keep a patient register from which TB patients can be selected. Thus, interpretation of our findings can only be restricted to the context of the formal health services. Although our findings are to a certain extend context bound, they may be relevant to other provinces in Indonesia with similar socio-economic, TB epidemiology and health system characteristics. Some specific findings may even hold in similar settings in other countries.

## Conclusion

Most TB patients in Jogjakarta took over a month to be diagnosed as TB patient by a DOTS facility after first symptoms appeared and consulted several providers. The factors that seemed to influence care seeking behavior most strongly were income and advice from household members or friends. Mild stigma existed but does not seem to play a major role in care seeking behavior. Raising community awareness of the availability of free high-quality TB services could lead to earlier diagnosis and treatment. However, taking into account the relatively large contribution of health providers in delaying diagnosis, engaging diverse health care providers in DOTS arguably would provide a greater leverage.

## Competing interests

The authors declare that they have no competing interests.

## Authors' contributions

NR, YM, Sh, Su, Pu, YS, CV and MVDW made substantial contributions to conception and design. NR, Sh, Su, Pu and YS collected the data. NR, YM, Sh, Su, Pu, YS, CV and MVDW made substantial contribution to analysis and interpretation of data. NR, YM, Sh, Su, Pu, YS, CV and MVDW have been involved in drafting the manuscript. NR, YM and MVDW have contributed to revising the manuscript critically for important intellectual content. NR, YM, Sh, Su, Pu, YS, CV and MVDW have given final approval of the version to be published.

## Pre-publication history

The pre-publication history for this paper can be accessed here:


